# Clinical and laboratory characteristics of Prader-Willi syndrome

**DOI:** 10.1186/1687-9856-2013-S1-P60

**Published:** 2013-10-03

**Authors:** Bui Phuong Thao, Vu Chi Dung, Nguyen Ngoc Khanh, Can Thi Bich Ngoc, Ngo Diem Ngoc, Dinh Thi Hong Nhung, An Thuy Lan, Nguyen Thi Mai, Nguyen Phu Dat, Nguyen Thi Hoan

**Affiliations:** 1 National Hospital of Pediatrics, Hanoi, Vietnam; 2 Hanoi Medical University, Hanoi, Vietnam

## 

Prader-Willi syndrome (PWS) is a complex multisystem genetic disorder due the lack of expression of paternally inherited imprinted genes on chromosome 15q11-13. Clinical presentation includes hypotonia, hyperphagia, obesity, hypogonadism, learning difficulty. We studied clinical and laboratory of patient diagnosed and treated in National Hospital of Pediatrics, Hanoi (NHP). This is descriptive study. We collected 26 patients diagnosed of PWS by FISH in NHP from December 2007 to April 2012 were recruited in the study. Male/female ratio was 6/1. Patients diagnosed before 5 years occupied 53.5%. 85.7% of patients were found to have hypotonia at age of 4.92.0 months. 86.4% of patients had hypherphagia at age of 20.711.1 months. In patients aged of > 2 years, height SDS was +8.74.7 SD compared to gender and age. The figure of BMI was +10.33 SD. 4/7 of patients aged ≥ 6 years had micropenis. 91.7% of patients had cryptorchidism. 4/24 of patients (14.3%) had type 2 diabetes mellitus. Based on clinical presentation, more PWS patients could be diagnosed and treated early.

